# Functional properties and adipogenesis inhibitory activity of protein hydrolysates from quinoa (*Chenopodium quinoa* Willd.)

**DOI:** 10.1002/fsn3.1052

**Published:** 2019-05-17

**Authors:** Zhenxing Shi, Yuqiong Hao, Cong Teng, Yang Yao, Guixing Ren

**Affiliations:** ^1^ Institute of Crop Science Chinese Academy of Agricultural Sciences Beijing China; ^2^ Laboratory of Biomass and Green Technologies Gembloux Agro‐Bio Tech, University of Liege Gembloux Belgium

**Keywords:** adipogenesis inhibitory activity, anti‐inflammation, functional properties, quinoa protein hydrolysates

## Abstract

The functional properties and adipogenesis inhibitory activity of quinoa protein hydrolysates, prepared using papain, pepsin, and pancreatin for 0, 30, 60, 90, and 120 min, were studied. For these three kinds of proteases, the solubility of the hydrolysates significantly increased with the increasing DH in pH range of 3–8, while the EAI and ESI of these hydrolysates significantly decreased during hydrolysis. The anti‐inflammatory activity of these protein hydrolysates was measured. All of these protein hydrolysates showed high anti‐inflammatory activity. However, there was no significant difference in anti‐inflammatory activity between protein hydrolysates and total protein from quinoa. These protein hydrolysates also inhibited lipid accumulation during differentiation within the range of concentrations of 0–1,600 μg/ml, which exerted no cytotoxicity toward 3T3‐L1 cells. The protein hydrolysates from quinoa prepared using pepsin for 120 min (PEP‐120) had the highest activity with an IC_50_ value of 786.58 μg/ml. Moreover, LC‐MS/MS analysis of PEP‐120 showed that five main bioactive peptides, which have been demonstrated to have ACE inhibitor, antioxidant, and antithrombotic activities, were present in PEP‐120. In addition, gene expression and Western blot analysis revealed that PEP‐120 suppressed the 3T3‐L1 cell differentiation through the peroxisome proliferator‐activated receptor γ (PPARγ) pathway.

## 1 INTRODUCTION

Obesity has been gaining attention due to its negative effect on physical fitness, which is often related to the excess intake of energy and lack of exercise. In obese individuals, the incidence of chronic metabolic disorders, including coronary, hypertension, type 2 diabetes, and even cancer, has been much higher than that in thinner people. Due to the awful side effects produced by diet pills on the market, the effort has been made to develop functional foods that have remarkable anti‐obesity effects (Després, Lemieux, & Prud'Homme, 2001).

Quinoa (*Chenopodium quinoa* Willd.), a staple food of people in Andes region of South America, is cultivated extensively in Peru and Bolivia. Recently, the nutritional value of quinoa has been considered to be superior to those of many cereals and milk (Aluko & Monu, 2003). Quinoa has been rapidly rediscovered as a functional food owing to its high protein content and protein quality (Vega‐Gálvez et al., 2010). Quinoa protein has been reported to have a balanced amino acid composition and various physiological activities, such as cholesterol‐lowering effect and α‐glycosidase inhibitory activities (Abugoch, Romero, Tapia, Silva, & Rivera, 2008; Meyer, Heinstein, Burnofradosevich, Delfel, & Mclaughlin, 1990; Takao et al., 2005). Studies have shown that the bioactive and functional properties of protein can be improved by enzymatic hydrolysis under controlled conditions (Aluko & Monu, 2003; Mendis, Rajapakse, Byun, & Kim, 2005). Protein hydrolysates from different plants, such as soybean and black rice, have been shown to have a strong anti‐obesity effect (Jang et al., 2008; So et al., 2015). Apart from the bioactive properties of quinoa, the characteristics of its protein hydrolysates can directly affect its functional properties and its function as a food ingredient (Kristinsson & Rasco, 2000). However, there is limited information on different hydrolysis conditions, adipogenesis inhibitory activity, and functional properties of protein hydrolysates from quinoa.

In this research, we are committed to learning more about protein hydrolysates from quinoa. The detail objectives were as follows: (a) to prepare protein hydrolysates from quinoa with different degree of hydrolyses (DHs) using three different proteinases, (b) to evaluate their functional properties, (c) to evaluate their adipogenesis inhibitory activity, and (d) to investigate the mechanism responsible for their adipogenesis inhibitory activity.

## 2 MATERIALS AND METHODS

### 2.1 Reagents

The 3T3‐L1 cells were purchased from the Shanghai Institutes of Biological Sciences, Chinese Academy of Sciences (Shanghai, China). Fetal bovine serum (FBS), Dulbecco's modified Eagle's medium (DMEM), dexamethasone (DEX), 1‐methyl‐3‐isobutylxanthine (IBMX), 3‐(4,5‐dimethylthiazol‐2‐yl)‐2,5‐diphenyltetrazolium bromide (MTT), and insulin were purchased from Sigma Chemical Co. Papain, pepsin, and pancreatin were obtained from Baiao Biochemistry Co. TRIzol reagent was purchased from Invitrogen Co. cDNA reverse transcription kit was purchased from Applied Biosystems. Antiperoxisome proliferator‐activated receptor γ (PPARγ), CCAAT/enhancer‐binding protein α (C/EBPα), adipocyte fatty acid binding protein (aP2), lipoprotein lipase (LPL), uncoupling protein‐2 (UCP‐2), and β‐actin antibody were obtained from Santa Cruz Biotechnology. Dimethyl sulfoxide (DMSO), ethanol, and other chemicals were of chromatographic or analytical reagent grade.

### 2.2 Production of protein hydrolysate from quinoa

Quinoa seeds were obtained from Shanxi Yilong Quinoa Co., Ltd, and quinoa protein was prepared through alkali extraction and acid precipitation (Yao, Cheng, & Ren, 2014). The protein hydrolysates were prepared by using enzyme hydrolysis methods reported previously with some modifications (Cao, Liu, Hou, & Liu, 2009). Briefly, 5 g of quinoa protein was immersed in 100 ml of phosphate‐buffered solution (PBS) with different pH values. For papain, pepsin, and pancreatin, the pH was adjusted to 7.0, 2.0, and 7.5, respectively. The hydrolysis was operated at pH 7.0, 50°C for papain; pH 2.0, 37°C for pepsin; and pH 7.5, 37°C for pancreatin for up to 120 min. At different time points (0, 30, 60, 90, and 120 min), the DHs were carried out according to the method of Klompong, Benjakul, Kantachote, and Shahidi (2007).

Then, the protein hydrolysates, which showed different DHs, were placed in a boiling water bath and incubated for 10 min, and the resulting hydrolysates were centrifuged for 15 min at 10,000 *g*. Finally, the supernatant was collected and freeze‐dried for the further use.

### 2.3 Assay of functional properties

#### 2.3.1 Solubility

The solubility of the protein hydrolysates was determined using the method of Wu, Hettiarachchy, and Qi (1998) with some modifications. To determine solubility, 10 mg of sample was dispersed in 10 ml deionized water at the pH range of 3.0–7.0. Then, the sample was stirred at ambient temperature for 30 min. After centrifugation (1,200 *g*, 10 min), protein solubility was determined using the Bradford method and calculated as:

solubility(%)=proteinconterntinsupernatant/totalproteincontentinsample×100

#### 2.3.2 Emulsifying properties

To determine the emulsifying properties, 2 ml of soybean oil and 6 ml of sample solution (0.1%, pH 3.0–7.0) were homogenized for 1 min (Wu et al., 1998). Fifty microliters of the emulsion was pipetted from the bottom of the container at 0 and 10 min after homogenization. Five milliliters of SDS solution (0.1%) was added and mixed. Then, the absorbance was read at 500 nm with distilled water as blank. The emulsifying activity index (EAI) and emulsion stability index (ESI) were calculated according to the following formula:

$$\text{EAI}\left({\text{m}}^{2}/\text{g}\right)=2T\left(A_{0}\times \text{dilution}\;\text{factor}/C\times \Phi \times 10,000\right)$$

*T* = 2.303; *A_0_* = the absorbance read at 0 min; the dilution factor is 100, *C*: weight of the sample/unit volume (g/ml) of the aqueous phase before emulsion formation; Ф = the oil volume fraction of the emulsion; ESI (min) = *A*_0_ × Δ*t*/Δ*A*Δ*t* = 10 min, Δ*A* = *A*_0_ − *A*_10_.

### 2.4 Anti‐inflammatory activity of the protein hydrolysates from quinoa

Anti‐inflammatory activity of protein hydrolysates from quinoa was measured. The RAW264.7 macrophage cells (Institute for Biological Science, Chinese Academy of Sciences) were cultured in RPMI 1640 medium (10% FBS, 1% streptomycin, and 1% penicillin) at 37°C in 5% CO_2_. After reproducing to 80%–90% confluence, the cells were seeded in a 96‐well plate and then treated with the samples at different concentrations. Two hours later, 10 μl LPS (20 µg/ml) was added into the 96‐well plate. Nitric oxide concentration was measured in the culture supernatant after 24 hr of co‐incubation using Griess reagent based on the standard curve of NaNO_2_.

### 2.5 Adipogenesis inhibitory activity in 3T3‐L1 cells

#### 2.5.1 Cell cultures

The 3T3‐L1 cells were cultured and maintained in DMEM (containing 10% FBS, 100 units/ml penicillin–streptomycin). The cells were maintained at 37°C with a humidified atmosphere of 5% CO_2_.

#### 2.5.2 Cytotoxic activity

The cytotoxic activities of the protein hydrolysates against the 3T3‐L1 cells were evaluated by the MTT assay (Kim, Bae, Ahn, Lee, & Lee, 2007). The cells were seeded into 96‐well plates (1 × 10^4^ cells/well) and maintained at 37°C in 5% CO_2 _for 24 hr. Then, the cells were treated with different concentrations of samples for 48 hr at 37°C in 5% CO_2_. Twenty microliters of the MTT solution (1 mg/ml) was added to each well and incubated at 37°C for 4 hr in the dark. After the MTT reagent was removed, 200 μl of DMSO was added to dissolve the formazan crystals, followed by shaking for 5 min. Then, the absorbance was measured at 570 nm through a microplate reader (Bio‐Rad).

#### 2.5.3 3T3‐L1 pre‐adipocyte differentiation

The effect of the protein hydrolysates on the differentiation of 3T3‐L1 cells was determined based on the method reported by Yim et al. (2011). The 3T3‐L1 cells were seeded (1 × 10^4^ cells/well) into 6‐well plates and cultured until confluent at 37°C in 5% CO_2_. After 48 hr, the cells were transferred to differentiation medium I DMEM supplemented with 0.5 mM IBMX, 10 μg/ml insulin, and 0.1 μM DEX and cultured for 48 hr again. Then, differentiation medium I was replaced by differentiation medium II, supplemented with 5 μg/ml insulin in DMEM; 48 hr later, the cells were treated with different concentrations of the samples which dissolved in differentiation medium II.

After 48 hr of treatment, the cells were washed with PBS and fixed with 10% formalin for 20 min. After washed with PBS twice again, the cells were stained with Oil Red O for 1 hr. Differentiated adipocytes were observed through an Olympus microscope. For quantification, the lipids in each well were extracted with 1 ml of isopropanol, and the absorbance was read at 492 nm (Kawai et al., 2007).

### 2.6 Characterization of peptides

Characterization of the peptides was carried out by Sangon Biotech. Co. Ltd. Briefly, the separation of the peptides was performed on an Eksigent nanoLC Ultra 2D system (AB SCIEX) using a ChromXP C18 column (120 Å, 3 μm, 7.5 mm × 150 mm) at 25°C with a mobile phase composed of a mixture of 0.1% formic acid and 2% acetonitrile in water (A) and 0.1% formic acid and 98% acetonitrile in water (B). The dried sample was dissolved in mobile phase A. The flow rate was 2 ml/min. The following gradient was used: 5%–35% B over 90 min. Mass spectrometry was performed on a Triple TOF 5600 system (AB SCIEX) according to the method of Chen et al. (2013). For information‐dependent acquisition, survey scans were conducted in 250 ms, and there were up to 35 product ion scans that could be collected when they exceeded a threshold of 150 counts/s with a 2^+^–5^+^ charge state. Based on the combination of MS and MS/MS spectra, the proteins could be successfully identified when they showed 95% or a higher confidence interval in the MASCOT V2.3 search engine (Matrix Science Ltd.). The identification of peptides was conducted according to the peptide database (http://www.uwm.edu.pl/biochemia/index.php/en/biopep).

### 2.7 Real‐time polymerase chain reaction and Western blot

#### 2.7.1 Real‐time polymerase chain reaction

The total mRNA was extracted with TRIZOL reagent and then reverse transcribed into complementary DNA (cDNA) using a high‐capacity cDNA reverse transcription kit. Real‐time polymerase chain reaction (RT‐PCR) was performed on a 7300 Real‐Time PCR System (Applied Biosystems) with β‐actin gene as the control to normalize the amount of the template in the PCR. All the primers used for real‐time PCR are listed in Table 1. The gene expression was calculated using the 2^−△△CT^ method.

**Table 1:** The primer sequence used for real‐time PCR

Gene name	Forward primer	Reverse primer	Accession no.
PPARγ	TTTTCAAGGGTGCCAGTTTC	AATCCTTGGCCCTCTGAGAT	NM_011146
C/EBPα	TTACAACAGGCCAGGTTTCC	GGCTGGCGACATACAGTACA	NM_007678
aP2	GGCCAAGCCCAACATGATC	CACGCCCAGTTTGAAGGAAA	NM_024406
LPL	CATCGAGAGGATCCGAGTGAA	TGCTGAGTCCTTTCCCTTCTG	NM_008509
β‐Actin	CCACAGCTGAGAGGGAAATC	AAGGAAGGCTGGAAAAGAGC	X03672

#### 2.7.2 Western blot

A previous method reported by Zhu et al. (2016) was used to perform the Western blot analysis. The cells were washed by PBS and lysed by ice‐cold RIPA lysis buffer. The supernatant was collected after centrifugation (12,000 *g*, 20 min, 4°C), separated by 12% polyacrylamide gel, and then transferred to a nitrocellulose membrane. After blocking for 2 hr at room temperature in 5% nonfat dry milk with 0.1% Tween‐20, the antibodies including anti‐PPARγ, aP2, C/EBPα, LPL, and β‐actin were added. Two hours later, the membrane was washed with 20 ml PBST for three times (each time period was 10 min) and then incubated with an HRP‐conjugated secondary antibody for 1 hr. After stringently washed with 20 ml PBST for three times, the signal was detected through a chemiluminescent apparatus (Thermo Fisher Scientific).

### 2.8 Statistics

Values were expressed as the means ± *SD*, and all of the above assays were repeated at least three times. One‐way analysis of variance (ANOVA) and Tukey's test were performed using SPSS (Statistical Package for Social Science) version 17.0. All graphical representations were conducted using SPAA 11.0. Statistical significance was set at *p* < 0.05.

## 3 RESULTS AND DISCUSSIONS

### 3.1 Hydrolysis of quinoa protein

**Figure 1:**
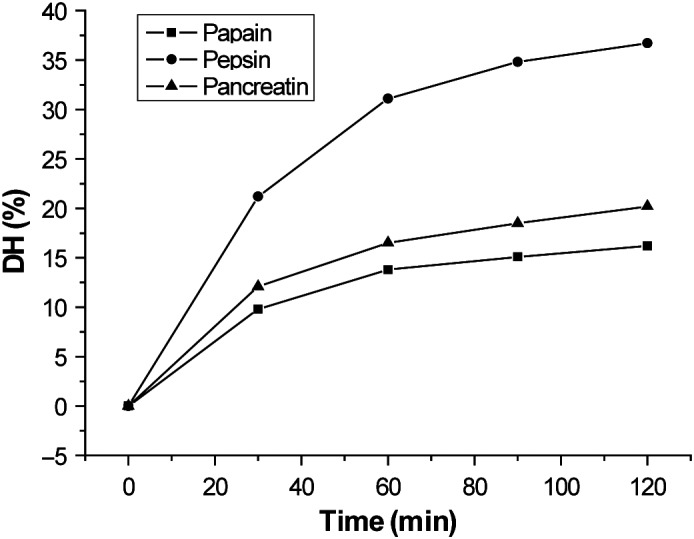
Degree of hydrolysis of quinoa protein. The hydrolysis was carried out with papain, pepsin, and pancreatin at 0, 30, 60, 90, and 120 min

The hydrolysis of quinoa protein was carried out using papain, pepsin, and pancreatin, respectively. As shown in Figure 1, the degree of hydrolysis of protein was increased over the time of 0–120 min. The protein was hydrolyzed rapidly within 30 min, but the hydrolysis rate became slower between 30 and 120 min. Besides, the degree of hydrolysis of protein by pepsin was significantly higher than degree of hydrolysis of protein by papain and pancreatin, and similar results have been observed by Montoya‐Rodríguez, Mejía, Dia, Reyes‐Moreno, and Milán‐Carrillo (2014). At the same time points, higher DHs were observed in proteins hydrolyzed using pepsin compared to those hydrolyzed using pancreatin (Himonides, Taylor, & Morris, 2011).

### 3.2 Analysis of functional properties

**Figure 2:**
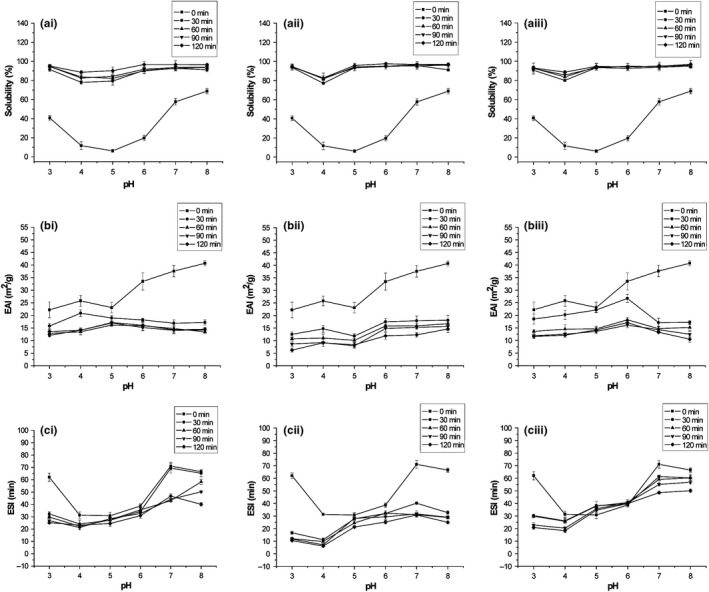
Solubility (a), EAI (b), and ESI (c) of protein hydrolysates from quinoa prepared using papain (I), pepsin (II), and pancreatin (III). Three independent experiments were performed, and the data are shown as mean ± *SD*. EAI, emulsifying activity index; ESI, emulsion stability index

The solubility, EAI, and ESI of the protein hydrolysates from quinoa prepared using papain, pepsin, and pancreatin for 0–120 min are presented in Figure 2. As shown in the pH range of 3–8, the solubility of all the hydrolysates was significantly higher (*p* < 0.05) compared to those of the control (0 min), suggesting that hydrolysis improved the solubility of quinoa protein. It has been reported that the generation of low molecular weight peptides and modification of polar and ionizable groups of the protein during hydrolysis lead to improved solubility of the protein (Turgeon & Gauthier, 1990). For the proteases of papain, pepsin, and pancreatin, the solubility of the hydrolysates increased with the increasing DH, which was consistent with the results of Klompong et al. (2007), and Klompong et al. (2007) showed that the fish protein hydrolysates, which exhibited high solubility, had high DHs. Additionally, Gbogouri, Linder, Fanni, and Parmentier (2010) obtained protein hydrolysates from salmon heads, which were enzymatically treated with Alcalase, and the results showed that the hydrolysates with high DH had the high solubility.

In this study, the EAI and ESI of these hydrolysates significantly decreased (*p* < 0.05) during the hydrolysis. It has been demonstrated that excessive hydrolysis leads to weak emulsifying activity and lower molecular weight or more hydrophilic peptides bring poor stability of the emulsion (Kristinsson & Rasco, 2000). However, the ESI of the hydrolysates prepared by pancreatin was exceptional in the pH range of 5 and 6, and was obviously higher than the control (0 min). It may be that more amphiphilic groups in the peptides were produced during hydrolysis with pancreatin.

### 3.3 Anti‐inflammatory activity analysis

**Figure 3:**
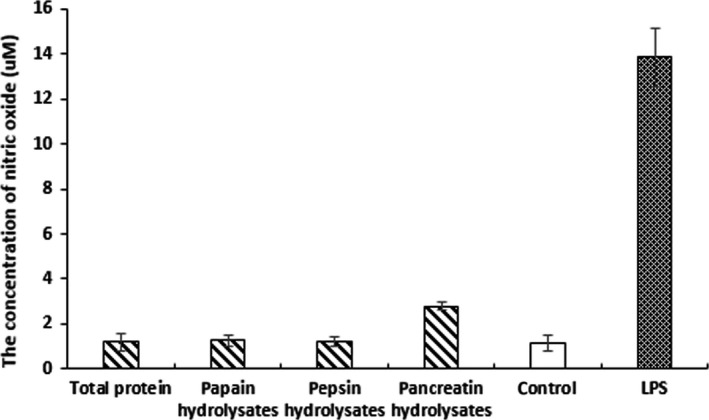
Anti‐inflammatory activity of protein hydrolysates from quinoa prepared using papain, pepsin, and pancreatin. The production of NO in RAW264.7 cells was inhibited by protein hydrolysates. Three independent experiments were performed, and the data are shown as mean ± *SD*

The anti‐inflammatory activity of quinoa protein hydrolysates was evaluated in the LPS‐stimulated immune reaction, which can stimulate phagocytic cells to produce signaling mediators, such as NO (Medvedev Et al., 2000). In this study, the release of NO was significantly inhibited by the protein hydrolysates from quinoa (Figure 3). Studies have shown that in vitro enzymatic hydrolysis has been applied commercially in larger volumes, which was used to produce the peptide with a multitude of beneficial metabolic effects, including the anti‐inflammatory activity (Cynthia, Stephen, & Chao‐Wu, 2018). However, we found that the total protein from quinoa also exhibited high anti‐inflammatory activity. There was no obvious difference in activity between total protein and protein hydrolysates.

### 3.4 Cytotoxicity analysis against 3T3‐L1 cells

**Figure 4:**
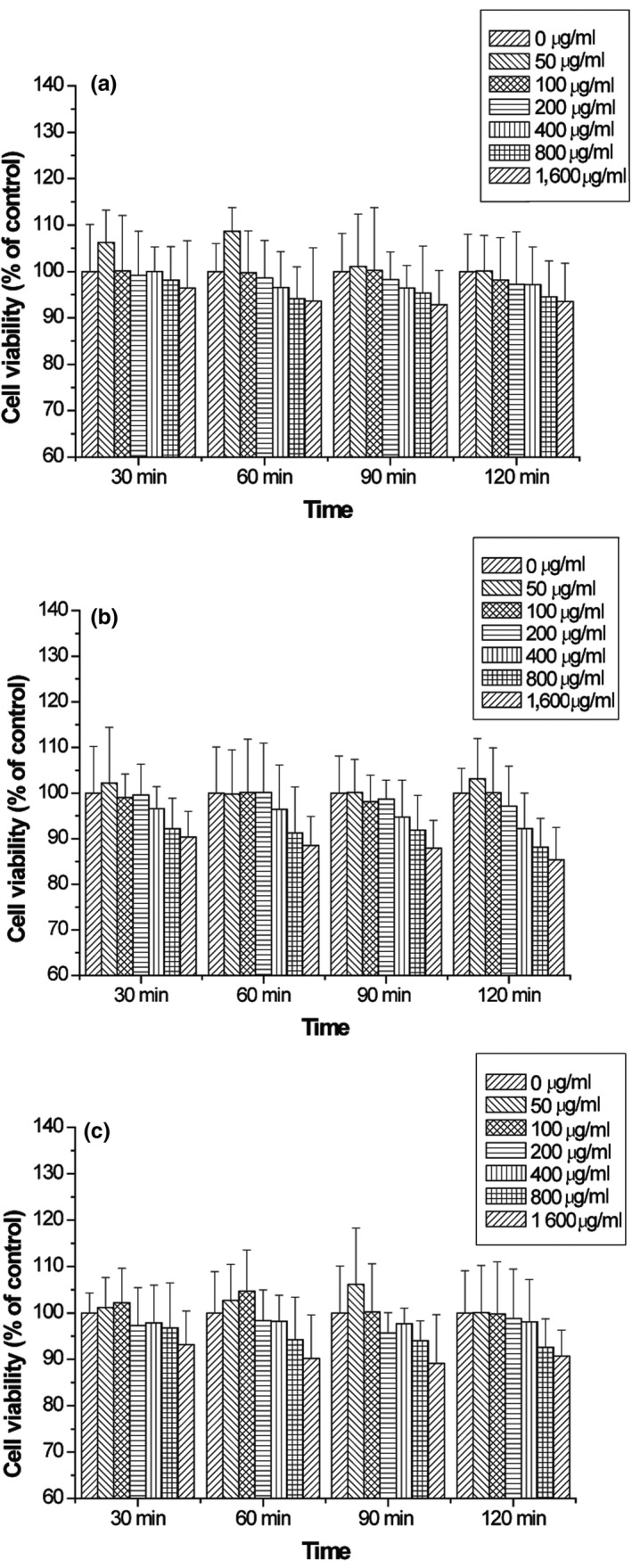
Cytotoxic effect of protein hydrolysates from quinoa hydrolyzed with papain (a), pepsin (b), and pancreatin (c) against 3T3‐L1 cells. Three independent experiments were performed, and the data are shown as mean ± *SD*

The cytotoxic activity of the protein hydrolysates from quinoa under different conditions was evaluated. The 3T3‐L1 pre‐adipocyte cells were treated with the samples at different concentrations of 0, 50, 100, 200, 400, 800, and 1,600 μg/ml. As a result, in the entire concentration range, there was no significant difference in the viability of 3T3‐L1 pre‐adipocytes, which were observed for papain, pepsin, and pancreatin hydrolysates (Figure 4a–c), suggesting that quinoa protein hydrolysate was not toxic to the 3T3‐L1 cells. Similar to this result, Kim et al. (2007) reported that black soybean hydrolysates did not induce cytotoxic responses at concentrations less than 10 mg/ml.

### 3.5 Adipogenesis inhibitory activity analysis

To investigate the adipogenesis inhibitory activity, the 3T3‐L1 adipocytes induced by insulin, DEX, and IBMX were treated with different samples for 48 hr. Within the range of concentrations of 0–1,600 μg/ml, the IC_50_ value of each sample was calculated. As it is shown in Table 2, a significant difference (*p* < 0.05) was observed in IC_50_ value of these samples, suggesting that both the type of enzyme used in hydrolysis and DH affected the adipogenesis inhibitory activity. Among them, the protein hydrolysates from quinoa hydrolyzed with pepsin exerted higher adipogenesis inhibitory activity, and as the DH increased, the IC_50_ value decreased (*p* < 0.05). Similarly, Tomita et al. (1991) found that bovine lactoferrin hydrolysates prepared with pepsin showed strong antibacterial activity, whereas hydrolysates prepared with papain were less active. In addition, Klompong et al. (2007) observed that the metal chelating activity of protein hydrolysates from yellow stripe trevally increased with increasing DH.

**Table 2:** Adipogenesis inhibitory activity of protein hydrolysates from quinoa hydrolyzed with papain, pepsin, and pancreatin

Enzyme type	Hydrolysis time (min)	IC50 (μg/ml)
Papain	30	nd
	60	nd
	90	1,209.28 ± 119.23a
	120	1,311.92 ± 110.56a
Pepsin	30	nd
	60	1,096.74 ± 89.33ab
	90	806.73 ± 79.94bc
	120	786. 58 ± 47.28c
Pancreatin	30	nd
	60	nd
	90	nd
	120	1,389.67 ± 96.79a

Note

Three independent experiments were performed, and the data are shown as mean ± *SD*.

“nd” means that the inhibition of sample was less than 50% at the highest concentration.

Tomita et al. (1991) have reported that peptic digests of protein could produce smaller peptides of less than 5 kDa, and lower molecular weight peptides exerted strong bioactivities. In this study, the protein hydrolysates from quinoa produced by pepsin for 120 min (PEP‐120) had the highest adipogenesis inhibitory activity, and their IC_50_ value was 786.58 μg/ml. It was demonstrated that peptides with different amino acid sequences could be generated depending on the protease specificity. Bioactivity has been affected by the amino acid compositions of the peptides, such as sequence PYY, which was known to have antioxidant activity, and sequence YL, which was known to have angiotensin‐converting enzyme (ACE) inhibitory activity (Yoshikawa et al., 2000; Kim et al., 2007; Montoya‐Rodríguez et al., 2014). Because of the highest adipogenesis inhibitory activity of PEP‐120, it was prepared and collected for further study.

### 3.6 Characterization of peptides of PEP‐120

The apparent suppressive effect of PEP‐120 on lipid accumulation in 3T3‐L1 cells during differentiation suggested its potential as an anti‐obese additive in functional foods. It is known that the amino acid sequence of peptides determines their bioactivities (Wu, Chen, & Shiau, 2003). Therefore, we next characterized PEP‐120 by LC‐MS/MS and bioinformatics analysis. The five main bioactive peptides, FGVSEDIAEKLQAKQDERGNIVL, AEGGLTEVWDTQDQQF, YIEQGNGISGLMIPG, AVVKQAGEEGFEW, and HGSDGNVF, were present in PEP‐120 (Table 3). After analysis, these peptides were associated with bioactivities such as the ACE inhibitor, antioxidant, and antithrombotic activities. Zemel and Miller (2004) analyzed the regulatory effect of dietary calcium and dairy on adiposity and obesity risk and founded that ACE inhibitors may contribute to the anti‐obesity effect.

**Table 3:** Bioactive peptides identified by LC‐MS/MS in protein hydrolysates from quinoa produced by pepsin for 120 min

Peptide sequence	Molecular mass (Da)	Bioactive sequence	Activity^a^
FGVSEDIAEKLQAKQDERGNIVL	2,567	FG, KL SE	ACE inhibitor Stimulator
AEGGLTEVWDTQDQQF	1,824	EG, GL	ACE inhibitor
YIEQGNGISGLMIPG	1,554	GL PG	ACE inhibitor Antioxidant, Antithrombotic
AVVKQAGEEGFEW	1,452	AG, EG	ACE inhibitor
HGSDGNVF	837	HG, DG	ACE inhibitor

Note

ACE inhibitor, angiotensin‐converting enzyme inhibitor.

a Identification of bioactive peptides was conducted using http://www.uwm.edu.pl/biochemia/index.php/en/biopep.

### 3.7 Suppressive effect of PEP‐120 on the PPARγ signal pathway in 3T3‐L1 cells

**Figure 5:**
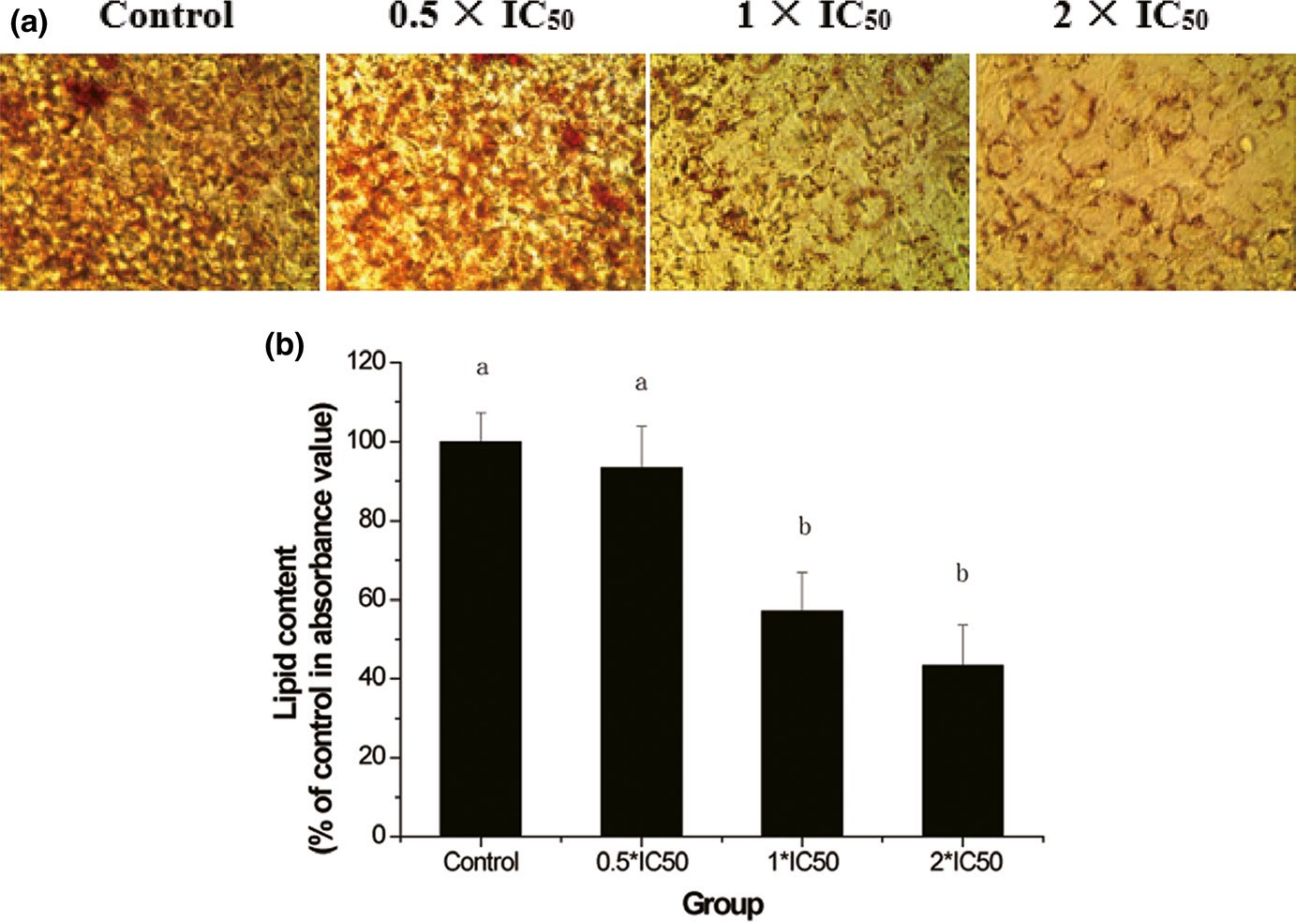
Effect of PEP‐120 on lipid accumulation in 3T3‐L1 cells. The mature cells were stained with Oil Red O (a), and the OD value (b) was measured to quantify intracellular lipid content. Three independent experiments were performed, and the data are shown as mean ± *SD*. Values do not share the same letter are significantly different (*p* < 0.05)

To further investigate the mechanism responsible for the adipogenesis inhibitory activity, 3T3‐L1 cells were treated with low (0.5 × IC_50_), medium (1 × IC_50_), and high (2 × IC_50_) doses of PEP‐120 during differentiation. Representative images of Oil Red O staining showed that PEP‐120 dose dependently inhibited lipid accumulation in 3T3‐L1 cells (Figure 5a), and quantitative data (Figure 5b) also demonstrated these results. Reductions of 6.60%, 42.91%, and 56.63% were observed for low, middle, and high doses of PEP‐120, respectively.

**Figure 6:**
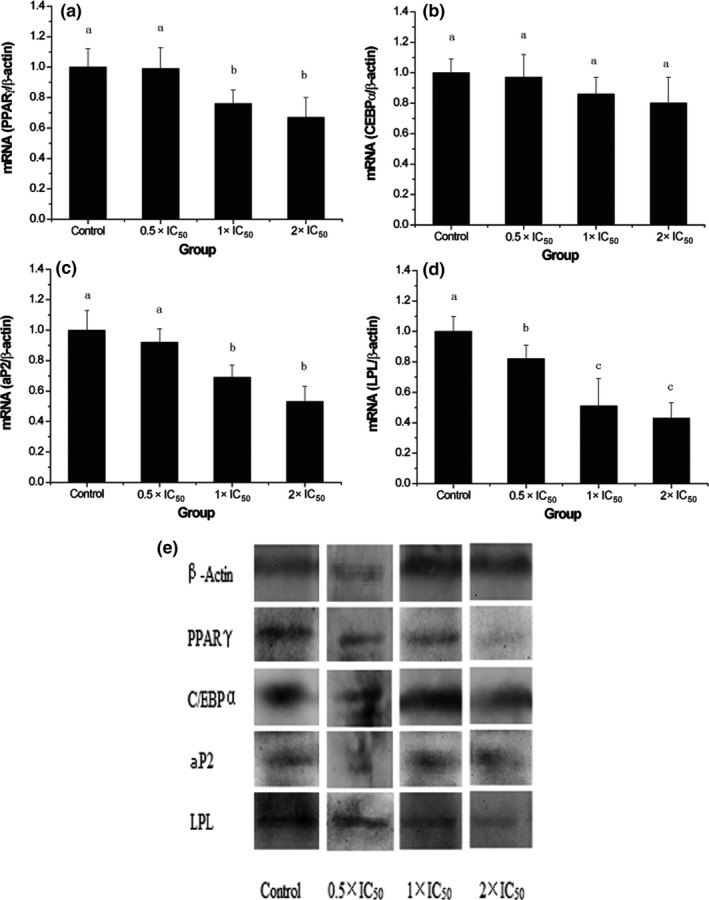
Effects of PEP‐120 on the mRNA expression of PPARγ (a), C/EBPα (b), aP2 (c), LPL (d), and their protein expression (e). Three independent experiments were performed, and the data are shown as mean ± *SD*. Values do not share the same letter are significantly different (*p* < 0.05). aP2, adipocyte fatty acid binding protein; ; C/EBPα, CCAAT/enhancer‐binding protein α; LPL, lipoprotein lipase; PPARγ, peroxisome proliferator‐activated receptor γ

Adipocyte differentiation is mediated by a series of programmed changes in gene expression (Ntambi & Youngcheul, 1989). Tontonoz, Hu, Graves, Budavari, and Spiegelman (1994) had reported that PPARγ was regarded as a master regulator of adipocyte differentiation and was abundantly expressed. Therefore, in this study, we are mainly concerned about the PPARγ pathway and measured the gene and protein expression of several adipogenic genes that play important roles in differentiation. As shown in Figure 6a,b, the PPARγ and C/EBPα expression levels were respectively reduced to 1.01%, 24.03%, and 33.33% and 3.10%, 14.01%, and 20.38% for low, medium, and high doses of PEP‐120. It has been demonstrated that PPARγ was required for adipocyte differentiation with the CCAAT/enhancer‐binding protein α (C/EBPα) (Rosen et al., 1999). The mRNA expression of aP2 and LPL was also examined, which has been used as the markers of adipocyte differentiation (Rosen et al., 1999). PEP‐120 significantly downregulated the gene and protein expression of aP2 and LPL (Figure 6c,d). AP2 and LPL are PPARγ target genes in fatty acid metabolism and can be regulated by PPARγ and C/EBPα (Rosen, Walkey, & Spiegelman, 2000). The adipocyte differentiation in vitro can be significantly suppressed when one of these factors was inhibited (Rosen et al., 1999). The protein expression levels of these factors were also examined (Figure 6e). Consistent with the mRNA expression, the protein expression levels were decreased.

## 4 CONCLUSIONS

In this study, we obtained the protein hydrolysates from quinoa using three different proteinases. The functional properties of quinoa protein hydrolysates varied with the enzyme type and hydrolysis time. These protein hydrolysates from quinoa could inhibit lipid accumulation during the differentiation of 3T3‐L1 cells. PEP‐120, protein hydrolysates from quinoa prepared using pepsin for 120 min, exhibited high solubility, and could significantly suppress 3T3‐L1 cell differentiation through the PPARγ pathway, which could be used as a functional food in the diets of obese patients in the future.

## CONFLICTS OF INTEREST

The authors declare that they have no conflicts of interest.

## ETHICAL STATEMENTS

The protocols and procedures were ethically reviewed and approved by the Chinese Academy of Agricultural Sciences. There was no human or animal testing in this study; ethics approval and consent to participate are not applicable to this manuscript.
